# Malpractice Suit

**Published:** 1881-07

**Authors:** 


MALPRACTICE SUIT.—It is highly gratifying to hear that an able and upright judge was so decided in his position, in supporting the right of the medical practitioner, as has so recently been done in the suit against Dr. Louis H. Sayre, of New York, for malpractice. A few more honest and manly rulings of that sort would go far toward deterring the designing and vicious among our patients, from depredating upon our means and reputation ; or attempting black-mail for real or fancied failure to meet all their wants and expectations in the medical or surgical treatment of their cases, or injuries. From the great zeal and energy displayed by council for the prosecution in some malpractice cases, it is much to be feared that lawyers are but too willing to enter upon the litigation of such cases, even if, in some of them at least, they may not be the incitors or instigators of the suits themselves. Of course no honorable attorney-at-law would be guilty of such nefarious conduct, knowing or even believing a case against a reputable medical man was destitute of real merit. But unfortunately the legal profession has its black sheep, as well as some other callings, and medical men should not be slow in honoring the judges of the law who sit in such cases, when the uprightness and ability displayed in Dr. Sayre's case is so clearly evident. We may revert to the matter of malpractice in a future issue.

